# Mining severe drug-drug interaction adverse events using Semantic Web technologies: a case study

**DOI:** 10.1186/s13040-015-0044-6

**Published:** 2015-03-25

**Authors:** Guoqian Jiang, Hongfang Liu, Harold R Solbrig, Christopher G Chute

**Affiliations:** Department of Health Sciences Research, Mayo Clinic, Rochester, MN USA

**Keywords:** Drug-drug Interaction, Adverse drug event, Data mining, Semantic web technology, Electronic medical records

## Abstract

**Background:**

Drug-drug interactions (DDIs) are a major contributing factor for unexpected adverse drug events (ADEs). However, few of knowledge resources cover the severity information of ADEs that is critical for prioritizing the medical need. The objective of the study is to develop and evaluate a Semantic Web-based approach for mining severe DDI-induced ADEs.

**Methods:**

We utilized a normalized FDA Adverse Event Report System (AERS) dataset and performed a case study of three frequently prescribed cardiovascular drugs: Warfarin, Clopidogrel and Simvastatin. We extracted putative DDI-ADE pairs and their associated outcome codes. We developed a pipeline to filter the associations using ADE datasets from SIDER and PharmGKB. We also performed a signal enrichment using electronic medical records (EMR) data. We leveraged the Common Terminology Criteria for Adverse Event (CTCAE) grading system and classified the DDI-induced ADEs into the CTCAE in the Web Ontology Language (OWL).

**Results:**

We identified 601 DDI-ADE pairs for the three drugs using the filtering pipeline, of which 61 pairs are in Grade 5, 56 pairs in Grade 4 and 484 pairs in Grade 3. Among 601 pairs, the signals of 59 DDI-ADE pairs were identified from the EMR data.

**Conclusions:**

The approach developed could be generalized to detect the signals of putative severe ADEs induced by DDIs in other drug domains and would be useful for supporting translational and pharmacovigilance study of severe ADEs.

## Introduction

Drug-drug interactions (DDIs) are a major contributing factor for unexpected adverse drug events (ADEs) [1]. A semantically coded knowledge base of DDI-induced ADEs with severity information is critical for clinical decision support systems and translational research applications. In particular, there is emerging interest in investigating genetic susceptibility of DDI-induced ADEs and developing genetic tests to identify all those at risk of ADEs prior to prescribing potentially dangerous medication [2,3], in which the severity information is essential for prioritizing the medical need to evaluate the potential impact of pharmacogenomics information in reducing ADEs [4]. However, few of knowledge resources cover severity information of ADEs.

While recognizing, explaining and ultimately predicting DDIs constitute a huge challenge for medicine and public health, informatics-based approaches are increasingly used in dealing with the challenge [5]. Semantic Web technologies provide a scalable framework for data standardization and data integration from heterogeneous resources. For instance, Samwald et al. [6] developed a Semantic Web-based knowledge base for query answering and decision support in clinical pharmacogenetics, in which three dataset components are integrated. In our previous and ongoing study, we developed a standardized knowledge base of ADEs known as ADEpedia (http://adepedia.org) leveraging Semantic Web technologies [7]. The ADEpedia is intended to integrate existing known ADE knowledge for drug safety surveillance from disparate resources such as Food and Drug Administration (FDA) Structured Product Labeling (SPL) [7], FDA Adverse Event Reporting System (AERS) [8], and the Unified Medical Language System (UMLS) [9].

The objective of the study is to develop and evaluate a Semantic Web-based approach for mining severe DDI-induced ADEs. We utilized a normalized FDA AERS dataset and performed a case study of three frequently prescribed cardiovascular drugs: Warfarin, Clopidogrel and Simvastatin. We extracted putative DDI-ADE pairs and their associated outcome codes. We developed a pipeline to filter the associations using ADE datasets from SIDER and PharmGKB. We also performed a signal enrichment using electronic medical records (EMR) data. We leveraged the Common Terminology Criteria for Adverse Event (CTCAE) grading system and classified the DDI-induced ADEs into the CTCAE in the Web Ontology Language (OWL).

## Background

### FDA Adverse Event Reporting System (AERS)

FDA AERS is a database that provides information on adverse event and medication error reports submitted to FDA [10]. By the definition of FDA, the “serious” means that one or more of the following outcomes were documented in the report: death (DE), hospitalization (HO), life threatening (LT), disability (DS), congenital anomaly (CA) and/or other (OT) serious outcome. In our previous study, we produced a normalized AERS dataset known as AERS-DM [11]. The dataset contains 4,639,613 unique putative Drug-ADE pairs in which the drugs are represented by RxNorm [12] codes and the putative ADEs are represented by MedDRA [13] codes. The data set also contains the unique ID number (known as ISR) for each corresponding AERS report, which is a primary link field between the AERS data file. We used the ISR field to identify the outcome codes of each AERS report. Table 1 shows the outcome code definitions in AERS database.

**Table 1:** **Outcome code definitions in AERS database**

Outcome code	Definition
DE	Death
LT	Life-Threatening
HO	Hospitalization - Initial or Prolonged
DS	Disability
CA	Congenital Anomaly
RI	Required Intervention to Prevent Permanent Impairment/Damage
OT	Other

### Common Terminology Criteria for Adverse Event (CTCAE)

CTCAE is a widely accepted, standard grading scale for adverse events throughout the oncology research community [14]. The current released version is CTCAE 4.0. This version contains 764 AE terms and 26 “Other, specify” options for reporting text terms not listed in CTCAE. Each AE term is associated with a 5-point severity scale. The AE terms are grouped by MedDRA Primary SOC classes. In the CTCAE, “Grade” refers to the severity of the adverse event (AE). The CTCAE displays Grades 1 through 5 with unique clinical descriptions of severity for each AE based on a general guideline. Table 2 shows the grade definitions in the CTCAE grading system.

**Table 2:** **Grade definitions in the CTCAE grading system**

Grade	Definition
Grade 1	Mild; asymptomatic or mild symptoms; clinical or diagnostic observations only; intervention not indicated.
Grade 2	Moderate; minimal, local or noninvasive intervention indicated; limiting age-appropriate instrumental ADL*.
Grade 3	Severe or medically significant but not immediately life-threatening; hospitalization or prolongation of hospitalization indicated; disabling; limiting self care ADL**.
Grade 4	Life-threatening consequences; urgent intervention indicated.
Grade 5	Death related to AE.

Note: Activities of Daily Living (ADL); *Instrumental ADL refer to preparing meals, shopping for groceries or clothes, using the telephone, managing money, etc.; **Self care ADL refer to bathing, dressing and undressing, feeding self, using the toilet, taking medications, and not bedridden.

### ADE datasets

SIDER (**SID**e **E**ffect **R**esource) is a public, computer-readable side effect resource that contains information on marketed medicines and their recorded adverse drug reactions [15]. The information is extracted from public documents and package inserts, in particular, from the US FDA Structured Product Labels (SPLs). The current version was released on October 17, 2012.

PharmGKB DDI-ADE Dataset is a database of DDI side effects based on FDA AERS reporting data [16], in which the confounding factors for prediction of the side effects are corrected through leveraging covariates in observational clinical data [17].

### Semantic Web technologies

The World Wide Web consortium (W3C) is the main standards body for the World Wide Web [18]. The goal of the W3C is to develop interoperable technologies and tools as well as specifications and guidelines to lead the web to its full potential. The resource description framework (RDF), web ontology language (OWL), and SPARQL (a recursive acronym for **S**PARQL **P**rotocol and **R**DF **Q**uery **L**anguage) specifications have all achieved the level of W3C recommendations, and are becoming generally accepted and widely used. RDF is a model of directed, labeled graphs that use a set of triples. Each triple is modeled in the form of subject, predicate and object. SPARQL is a standard query language for RDF graphs. OWL is a standard ontology language used for ontology modeling.

## Methods

We utilized a normalized AERS dataset known as AERS-DM that was produced in a previous study [11]. The dataset contains 4,639,613 unique putative Drug-ADE pairs in which the drugs are represented by RxNorm codes and the putative ADEs are represented by MedDRA codes. The AERS-DM dataset is organized in two database files in the Tab Separated Values (TSV) format and accessible at: http://informatics.mayo.edu/adepedia/index.php/Download.

**Figure 1:**
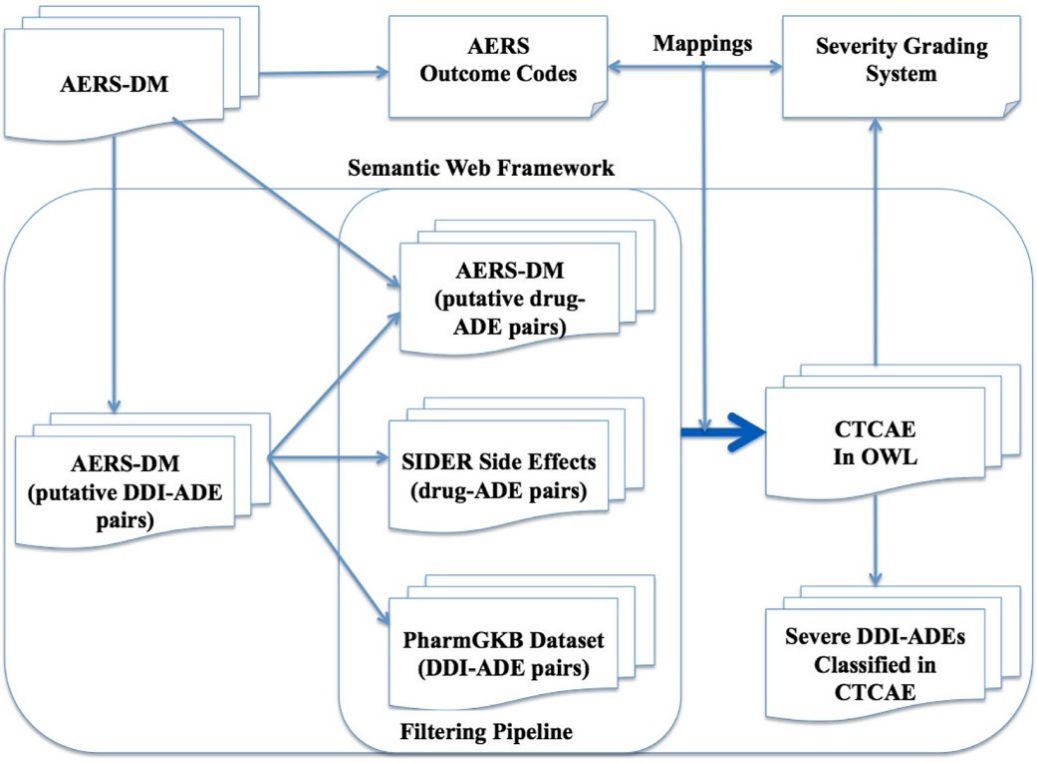
**System architecture.**

Figure 1 shows the system architecture of our approach. We first extracted a subset of putative DDI-ADE pairs (in which only two drugs are listed on a report) with their associated outcome codes from original AERS-DM dataset.

Second, we developed a filtering pipeline that comprises three datasets. The first dataset is a subset of original AERS-DM in which only one drug is listed on a report. This dataset was used to build a knowledge base of severe ADEs in a previous study. The second dataset is the SIDER 2 dataset. Table 3 shows a list of drug-ADE pair examples from the dataset, in which drug names are coded in STICH ID (http://stitch.embl.de) and ADE names are coded in MedDRA. We excluded the putative DDI-ADE pairs based on the Drug-ADE pairs of the two datasets. The filtering would ensure that the reported ADEs could not be explained by a single drug effect. The third dataset is a PharmGKB dataset that is used as “silver” standard. Table 4 shows a list of DDI-ADE examples from the dataset, in which drug names are coded in STICH ID and ADE names are coded in UMLS Concept Unique Identifiers (CUIs).

**Table 3:** **A list of Drug-ADE examples from SIDER dataset, in which drug names are coded in STICH ID and ADE names are coded in MedDRA**

stitch_id1	stitch_id2	UMLS_con cept_id	Drug_name	side_effect_name	MedDRA_conscept_type	UMLS_concept_id	MEDDRA_side_effect_name
−100003914	−39468	C0038454	Levobunolol	cerebrovascular accident	LLT	C0038454	Cerebrovascular accident
−100003914	−39468	C0038454	Levobunolol	cerebrovascular accident	PT	C0038454	Cerebrovascular accident
−100003914	−39468	C0015230	Levobunolol	rash	LLT	C0038454	Rash
−100003914	−39468	C0015230	Levobunolol	rash	PT	C0015230	Rash
−100003914	−39468	C0015230	Levobunolol	rash	PT	C0015230	Dermatitis
−100003914	−39468	C0033377	Levobunolol	ptosis	LLT	C0011603	Ptosis
−100003914	−39468	C0033377	Levobunolol	ptosis	PT	C0033377	Eyelid ptosis
−100003914	−39468	C0033377	Levobunolol	ptosis	PT	C0005745	Uterovaginal prolapse
−100003914	−39468	C0030554	Levobunolol	paresthesia	LLT	C0156353	Paraesthesia
−100003914	−39468	C0030554	Levobunolol	paresthesia	PT	C0030554	Paraesthesia
−100003914	−39468	C0006266	Levobunolol	bronchospas	LLT	C0006266	Bronhospasm
−100003914	−39468	C0006266	Levobunolol	bronchospas	PT	C0006266	Bronhospasm
−100003914	−39468	C1145670	Levobunolol	respiratory failure	LLT	C1145670	Respiratory failure
−100003914	−39468	C1145670	Levobunolol	respiratory failure	PT	C1145670	Respiratory failure
−100003914	−39468	C0027424	Levobunolol	nasal congestion	LLT	C0027424	Nasal congestion
−100003914	−39468	C0027424	Levobunolol	nasal congestion	PT	C0027424	Nasal congestion
−100003914	−39468	C0023380	Levobunolol	lethargy	LLT	C0023380	Lethargy
−100003914	−39468	C0023380	Levobunolol	lethargy	PT	C0023380	Lethargy
−100003914	−39468	C0947912	Levobunolol	myasthenia	LLT	C0947912	Mysathenia
−100003914	−39468	C0947912	Levobunolol	myasthenia	PT	C0151786	Muscular weakness

**Table 4:** **A list of DDI-ADE examples from PharmGKB dataset, in which drug names are coded in STICH ID and ADE names are coded in UMLS CUI**

stitch_id1	stitch_id2	drug1	drug2	event_umls_id	event_name
CID000000085	CID000000206	carnitine	galatose	C0004623	Bacterial infection
CID000000085	CID000000206	carnitine	galatose	C0015967	body temperature increased
CID000000085	CID000000206	carnitine	galatose	C0018932	haematochezia
CID000000085	CID000000206	carnitine	galatose	C0020433	Bilirubinaemia
CID000000085	CID000000206	carnitine	galatose	C0022346	icterus
CID000000085	CID000000206	carnitine	galatose	C0026946	fungal disease
CID000000085	CID000000206	carnitine	galatose	C0030305	panreatitis
CID000000085	CID000000206	carnitine	galatose	C0040034	thrombpcytopenia
CID000000085	CID000000206	carnitine	galatose	C0085605	Hepatic failure
CID000000085	CID000000206	carnitine	galatose	C0151766	Abnormal LFTs
CID000000085	CID000000206	carnitine	galatose	C0243026	sepsis
CID000000085	CID000000271	carnitine	galatose	C0002792	anaphylactic reaction
CID000000085	CID000000271	carnitine	galatose	C0002871	anaemia
CID000000085	CID000000271	carnitine	galatose	C0002962	angina
CID000000085	CID000000271	carnitine	galatose	C0004238	AFIB
CID000000085	CID000000271	carnitine	galatose	C0010054	arteriosclerotic disease
CID000000085	CID000000271	carnitine	galatose	C0010200	Cough
CID000000085	CID000000271	carnitine	galatose	C0012833	dizziness
CID000000085	CID000000271	carnitine	galatose	C0013404	Difficulty breathing
CID000000085	CID000000271	carnitine	galatose	C0015802	femur fracture

Third, we converted all the datasets used in this study into the Semantic Web RDF format and loaded them into an open source RDF store known as 4store [19]. We established a SPARQL endpoint that provides standard query services against the RDF store. And then we developed the extraction and filtering algorithms using Java-based Jena ARQ APIs [20].

Third, to enrich the signals of the DDI-induced ADEs, we used the NLP-processed EMR data of a cohort of 138 k patients with health home care provided by Mayo Clinic Rochester where medications and problems have been extracted and normalized to RxNorm codes and the UMLS concepts from the current medication and problem list sections of clinical notes using MedXN and MedTagger (http://www.ohnlp.org/). For each DDI-induced ADE triples (D1, D2, P), we obtained the number of patients who are administrated with any of the two drugs or both (i.e., N(D1), N(D2), and N(D1,D2)) and the number of patients with putative ADEs (i.e., N(D1,P), N(D2,P), and N(D1,D2,P) after taking the drugs. An occurrence of problem P is considered as putative ADE if it happens within 36 days of drug administration [17] and there is no occurrence of P in the EMR before the drug administration. We then developed the following metric to measure the signal enrichment of DDI-induced ADE:

$$ Score\left(D1,D2,P\right)=lo{g}_2\left(\raisebox{1ex}{$\frac{N\left(D1,D2,P\right)}{N\left(D1,D2\right)}$}\!\left/ \!\raisebox{-1ex}{$ max\Big(\frac{N\left(D1,\;P\right)}{N(D1)},\;\frac{N\left(D2,P\right)}{N(D2)}$}\right.\right). $$

**Figure 2:**
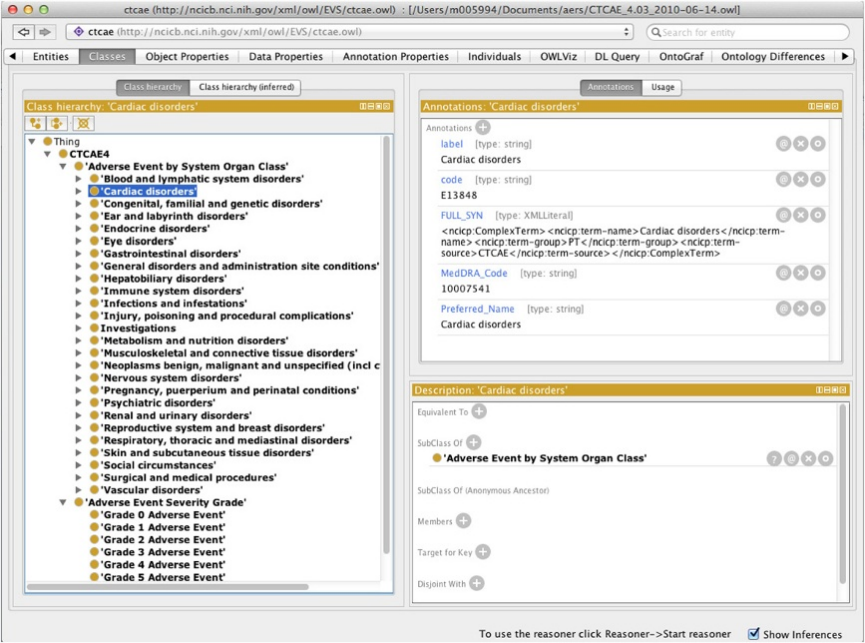
**The categories and severity grades of CTCAE classification in a Protégé 4 environment.**

Finally, we developed the mappings between AERS outcome codes and CTCAE grades and classified the filtered DDI-ADEs into the CTCAE. We asserted that DE in AERS corresponds to Grade 5 in CTCAE; LT corresponds to Grade 4; the rest of outcome codes (HO, DS, CA, RI and OT) correspond to Grade 3. In this study, we utilized the CTCAE version 4.0 [14] rendered in OWL format. Figure 2 shows a screenshot of a Protégé4 environment displaying the categories and severity grades in CTCAE classification.

## Results

We were able to extract a set of putative DDI-ADE pairs and their associated outcome codes for the three target drugs: Warfarin, Clopidogrel and Simvastatin from normalized AERS-DM dataset. We then filtered the putative DDI-ADE pairs using the filtering pipeline based on three datasets. Table 5 shows the number of filtered DDI-ADE pairs for each target drug. In total, 601 pairs were filtered. Of them, 61 pairs are classified in Grade 5, 56 pairs in Grade 4 and 484 pairs in Grade 3. Table 6 shows a list of filtered DDI-ADE pair examples for the drug “Simvastatin”, in which, drugs are coded in RxNorm RxCUIs and ADEs are coded in MedDRA codes.

**Table 5:** **The number of filtered DDI-ADE pairs for three drugs**

Drug	Number of DDI-ADE Pairs		
	Grade 5	Grade 4	Grade 3
Warfarin	32	11	157
Clopidogrel	17	29	166
Simvastatin	12	16	161
Total	61	56	484

**Table 6:** **A list of filtered DDI-ADE pairs for the drug “Simvastatin” classified by CTCAE grades**

CTCAE grade	AERS outome code	Drug code by RxCUI	Drug name	Drug code by RxCUI	Drug name	ADE code by MedDRA	ADE name
Grede 5	DE	36567	Simvastatin	1191	Aspirin	10002906	Aortic stenosis
Grede 5	DE	253198	Risiglitazone maleate	36567	Simvastatin	10006580	Bundle branch block left
Grede 5	DE	36567	Simvastatin	203160	Losartan Potassium	10007515	Cardiac arrest
Grede 5	DE	36567	Simvastatin	1191	Aspirin	10010071	Coma
Grede 5	DE	253198	Risiglitazone maleate	36567	Simvastatin	10012689	Diabetic retinoathy
Grede 4	LT	36567	Simvastatin	203029	Tegretol	10002948	Aphasia
Grede 4	LT	36567	Simvastatin	203029	Tegretol	10003119	Arrhythmia
Grede 4	LT	253198	Amiodarone hydrochloride	316675	Simvastatin 80 MG	10006002	Bone pain
Grede 4	LT	36567	Simvastatin	225807	exelon	10007515	Cardiac arrest
Grede 4	LT	36567	Simvastatin	203029	Tegretol	10012455	Dematitis exfoliative
Grede 3	DS	36567	Simvastatin	1191	Aspirin	10012455	Dematitis exfoliative
Grede 3	DS	36567	Simvastatin	190465	Viagra	10018429	Glucose tolerance impaired
Grede 3	DS	36567	Simvastatin	83367	Atorvastatin	10020765	Hypersomia
Grede 3	DS	36567	Simvastatin	35296	Ramipril	10050295	Intervertebral disc protrusion
Grede 3	DS	253198	Gemfibrozil 600 MG	316675	Simvastatin 80 MG	10000486	Acidosis

For the signal enrichment using the EMR data, we found that, there are 89 drug pairs prescribed concomitantly in 9.5 k patients, accounting for 6.9% of all patients in the EMR dataset we used. Out of 601 putative DDI-ADE pairs, the signals of 59 (D1, D2, P) pairs were identified. Table 7 shows the detailed statistics of those pairs occurred in no less than five patients.

**Table 7:** **A list of putative DDI-ADE pairs signaled in the EMR data**

D1 (RxCUI)	D2(RxCUI)	P (MedDRA)	ADE Name	N(D1)	N(D2)	N(D1,D2)	N(D1,P)	N(D2,P	N(D1,D2,P)	Score(DI,D2,P)
Aspirin (1191)	Simvastatin (36567)	10002906	Aortic stenosis	38149	7494	2926	104	34	15	4.991
Zocor (196503)	Simvastatin (36567)	10038428	Renal disorder	10894	7494	1472	40	56	7	4.550
Simvastatin (36567)	atorvastatin (83367)	10028417	Myasthenia gravis	7494	2841	828	42	10	5	4.409
Warfarin (11289)	Digoxin (3407)	10013887	Dysathria	6330	1927	641	43	7	6	4.36
Aspirin (1191)	Simvastatin (36567)	10015090	Epistaxis	38149	7494	2926	126	28	9	4.257
gabapentin (25480)	Simvastatin (36567)	10019245	Hearing impsored	4683	7494	280	35	70	5	3.935
Plavix (174742)	Simvastatin (36567)	10017955	Gastrointestinal heamorrhage	4769	7494	642	54	42	9	3.88
Aspirin (1191)	clopidogrel (32968)	10037423	Pulmunary oedema	38149	1436	1291	142	8	8	3.338
Aspirin (1191)	clopidogrel (32968)	10005191	Blister Dyspnoea exertion	38149	1436	1291	135	9	7	3.048
Amlodipine (17767)	Simvastatin (36567)	10013971	Dyspnoea exertional	2786	7494	561	62	89	11	2995
Aspirin (1191)	Simvastatin (36567)	10047924	Wheezing	38149	7494	2926	354	73	27	2.969
Lantus (261551)	Simvastatin (36567)	10012680	Diabetic neuropathy	1883	7494	329	39	20	5	2.63
Aspirin (1191)	clopidogrel (32968)	10038428	Renal disorder	38149	1436	1291	175	9	6	2.452
Lantus (261551)	clopidogrel (32968)	10040882	Skin lesion	38149	1436	1291	269	21	16	2.024
Aspirin (1191)	clopidogrel (32968)	10046555	Urinary retention	38149	1436	1291	292	16	11	1.757
Aspirin (1191)	clopidogrel (32968)	10061623	Adverse drug reaction	38149	1436	1291	368	20	15	1.549
Simvastatin (36567)	Norvasc (58927)	10017076	fracture	7494	3416	318	139	59	6	1.219

D1 - drug1, D2 - drug 2, P - problem, N – number, and Score – enrichment score.

**Figure 3:**
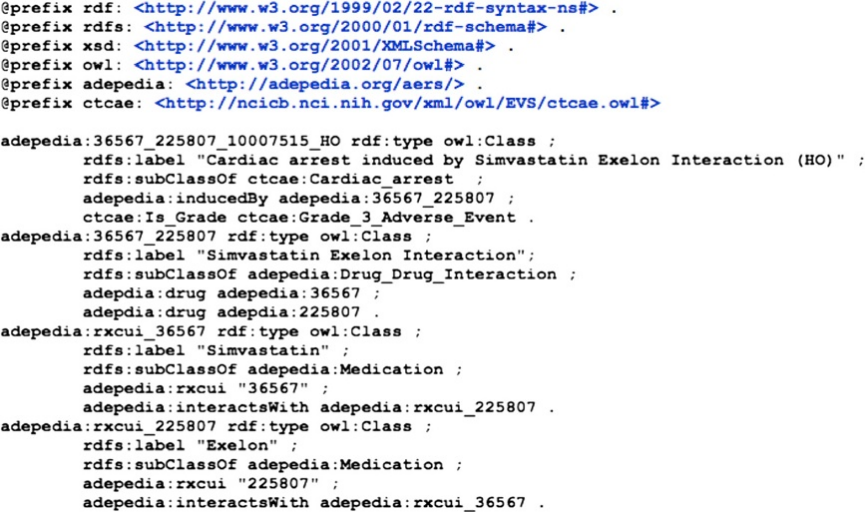
**The OWL representation of an example DDI-ADE.**

For integrating the filtered DDI-ADE pairs with the CTCAE, we produced an OWL rendering for each pair, asserting the filtered DDI-ADEs under AE terms in CTCAE (see Figure 3 for an example).

## Discussion

In a previous study, we used a similar Semantic Web-based approach to build a knowledge base of severe ADEs using the FDA AERS reporting data [8]. In this study, we focused on mining the DDI-induced ADEs and their severity information, and configured the filtering pipeline differently using a collection of ADE datasets. The standardization of ADE datasets is essential for enabling interoperability and comparability among heterogeneous data sources. We used a normalized AERS dataset, in which the drug names are normalized using standard drug ontologies RxNorm and NDF-RT and the ADEs are normalized using MedDRA, whereas the datasets from SIDER and PharmGKB used STITCH compound IDs to code drug names and used UMLS CUIs to code ADEs. Apparently, the solid mappings between RxNorm codes and STITCH IDs would be required in future, which will be part of our research efforts in constructing a standardized drug and pharmacological class network [21].

We also tested the signals of putative DDI-ADE pairs filtered by the pipeline using a large EMR data. We were able to detect some strong signals indicated by the enrichment score as illustrated in Table 7. This would potentially provide a very useful tool for the knowledge-driven detection of the DDI-induced ADEs from the EMR, though a rigorous patient chart review with a panel of clinicians would be needed in future to verify the signals to establish the causality of the drug-drug interaction.

For measuring the severity of ADEs, we used the CTCAE severity grading system. We found that the AERS outcome codes used to record serious patient outcomes in the AERS reporting data correspond well to the CTCAE Grades 3 to 5. Semantic Web OWL rendering of the DDI-ADE dataset provides seamless integration with the CTCAE itself, enabling a standard infrastructure for automatic classification of ADEs based on the severity conditions specified in the CTCAE.

There are several limitations in this study. First, we used the logic that a putative DDI-ADE combination is extracted if there exists an AERS report involving two drugs and the ADE. We understand that the AERS reports themselves do not make it easy to report concomitant drugs and these are known to be under-reported. This means the putative DDI-ADE pairs extracted in this study only reflect a portion of all DDI interactions and should not be considered as a comprehensive list. Second, the PharmGKB “silver standard” itself contains signals that have not been validated for causality. This is part of the reasons why we introduce the EMR-based signal enrichment metric in this study. Third, some signals identified from EMR data may not be valid and further rigorous validation approach will be needed in future to filter them out.

## Conclusions

In summary, we developed a Semantic Web-based approach to mine severe DDI-induced ADEs. The dataset produced in this study will be publicly available from our ADEpedia website (http://adepedia.org). The approach developed could be generalized to detect the signals from EMR for putative severe ADEs induced by DDIs in other drug domains and would be useful for supporting translational and pharmacovigilance study of severe ADEs.

## Consent

Informed consent of the use of EMRs for general research was provided by each subject with charts being included in the study. The study was approved by the Institutional Review Committee of the Mayo Clinic as Exempt (Mayo IRB Number: 12-009059).
